# Study on the Morphological Distribution and Modeling Methods of River Particles in Upstream and Downstream Sections

**DOI:** 10.3390/ma17215290

**Published:** 2024-10-30

**Authors:** Zhengbo Hu, Junhui Zhang, Xin Tan, Hao Yang

**Affiliations:** 1 National Engineering Laboratory of Highway Maintenance Technology, Changsha University of Science & Technology, Changsha 410114, China; zjhseu@csust.edu.cn (J.Z.); hyang_cityu@163.com (H.Y.); 2 College of Civil Engineering, Hunan University, Changsha 410082, China; xintan@hnu.edu.cn

**Keywords:** voronoi tessellation, abrasion process, particle morphology, discrete element analysis

## Abstract

This study investigates the morphological evolution of river particles and their mechanical behavior during sediment transport. River particles exhibit distinct shape differences between upstream and downstream sections, with particles becoming progressively rounded downstream. The rounding process is quantitatively described using morphological indices. The analysis reveals upstream particles are more angular, while downstream particles become increasingly rounded due to erosion and abrasion, modeled by a unified abrasion function. The Loop subdivision method effectively simulates this gradual rounding process. Additionally, the Discrete Element Method (DEM) calculates the natural angle of repose for particles with varying erosion levels, showing angles ranging from 38.2° for angular particles to 34.4° for rounded particles, closely matching field observations. The numerical results effectively demonstrate the interlocking effect caused by particle morphology. This research enhances the understanding of sediment transport dynamics and provides a robust framework for modeling particle shape evolution.

## 1. Introduction

River rocks, commonly characterized by their smooth and rounded shapes, are a fascinating outcome of natural geological processes, resulting from prolonged exposure to various erosional forces, primarily physical erosion driven by wind, water, ice, and gravity. As rocks are transported from their source at mountain tops down to river valleys, they undergo a series of transformative stages that gradually alter their shape and texture [1,2,3]. The process begins with the breakdown of source rocks due to weathering, which creates natural fractures, and these fragmented rocks then embark on a journey downhill, where they are subject to continuous tumbling and collisions as they move along riverbeds, causing them to lose their sharp edges through abrasion and eventually adopt a more rounded and ellipsoidal shape [4,5,6]. Over extended periods, which can span tens of millions of years, the rocks continue to be polished by sediment as they are carried downstream, and this abrasion and smoothing process results in the familiar polished appearance of river rocks [7]. Ultimately, the rocks may be reduced to pebbles and even finer sediments, contributing to the ongoing cycle of erosion within the river system. The diversity in particle morphology leads to significant differences in their mechanical properties, making it a research focus to accurately simulate these variations in modeling [8,9].

Research has shown that there are three primary mechanisms of particle attrition during sediment transport, ordered by increasing energy: frictional abrasion, chipping, and fragmentation [10,11]. In all cases, fragmentation produces irregular and sharp particles. Domokos [12] found that rock fragments formed through both slow weathering and rapid breakage exhibit characteristics determined by the geometry of brittle fractures. Giuseppe Buscarnera [13] proposes a new generation of constitutive laws for crushable granular continua by incorporating multiple grain shape descriptors into Continuum Breakage Mechanics (CBM) to account for the evolving particle morphology during comminution, providing insights into the feedback between particle shape. Additionally, rotating drum experiments simulating impact abrasion in pyroclastic flows revealed similar curves, suggesting that erosion mass can be inferred solely from shape data, allowing for the estimation of the transport distance of rounded pebbles found on Martian alluvial fans [14]. The ubiquity of rounding in fluvial, coastal, and aeolian environments provides qualitative evidence of the similarity in particle attrition dynamics due to collisions during sediment transport [1,2,15]. Furthermore, some studies have described the morphological evolution of particle rounding through functions [16,17] and validated their applicability via experiments [15,18]. While these theoretical analyses and laboratory studies offer valuable insights, research on numerical simulations remains relatively limited. Although researchers have successfully reconstructed particle shapes using ellipsoids, superellipsoids, spherical harmonics, and laser scanning techniques, there is still a lack of modeling approaches capable of accurately describing the process of particle rounding. Recent studies [19] have addressed particle abrasion and its influence on mechanical properties, but detailed morphological comparisons with real particles are still missing.

This study introduces a novel modeling approach for simulating the particle abrasion process, which is validated through comparisons of macroscopic and microscopic morphological indices with actual particles. Additionally, the mechanical effects of particle morphology are examined using both field observations and the Discrete Element Method (DEM).

## 2. Modeling Method of Particles

**Figure 1:**
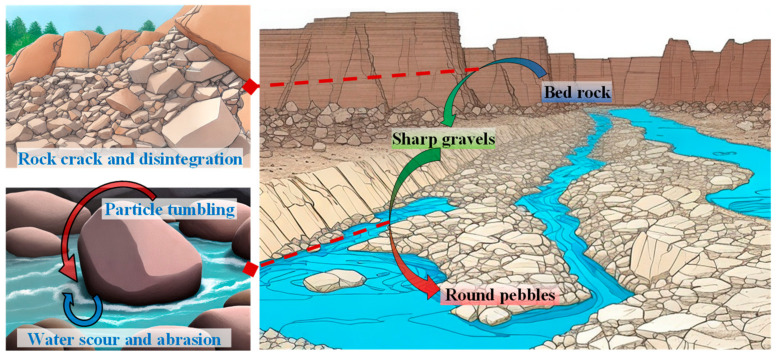
Diagram of the Particle Fracture and Abrasion Process.

As shown in Figure 1, bedrock in nature undergoes cracking and disintegration due to both internal and external factors. Typically, the particles formed by these processes are sharp and angular. However, under the influence of water flow, the particles are subjected to rolling and abrasion, gradually smoothing as they are transported downstream. This leads to more rounded shapes in the lower reaches of the river. In this section, we will introduce the rock fracture process and the corresponding abrasion modeling method.

### 2.1. Simulation of Bedrock Fracture Process

The simulation of particle shape randomness and multifacetedness is crucial in studying rock fragmentation, as it provides insights into rock mass behavior under various external and internal conditions. Both 2D and 3D modeling methods accurately capture the intricate shapes and interactions of these particles. In numerical simulations, rock masses are often assumed to fracture along Voronoi boundaries under external loads and internal stresses, resulting in smaller fragments that form complex structures [20,21]. Voronoi polyhedra are widely used in rock mechanics, particularly for simulating rock fracture and crack propagation. Numerical models like the Continuum Voronoi Block Model (CVBM) have shown strong accuracy in replicating laboratory tests, including Brazilian, unconfined compression, and triaxial tests [22,23]. Additionally, real-time rigid body fracture simulations based on Voronoi diagrams offer realistic results by combining energy conservation and material mechanics to calculate fracture zones and fragment velocities.

**Figure 2:**
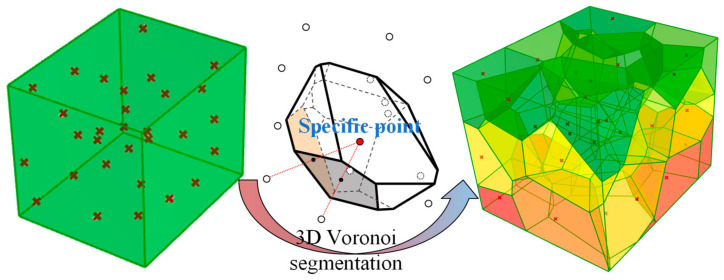
Three-dimensional Voronoi Tessellation within a Cube Diagram.

In Figure 2, the generation of 30 randomly positioned points within a three-dimensional cube illustrates how Voronoi polyhedra can represent the shapes of rock masses after fracturing. Each adjacent pair of points is connected by a line segment, and the perpendicular bisecting plane of each segment is constructed. The stones are assumed to fracture along the boundaries between these polyhedra, ultimately dividing the original cube into 30 Voronoi polyhedra. Researchers typically consider these Voronoi polyhedra as the shapes of rock masses after fracturing, demonstrating the significant potential of Voronoi polyhedra in simulating rock fracture processes.

### 2.2. Particle Abrasion Method

**Figure 3:**
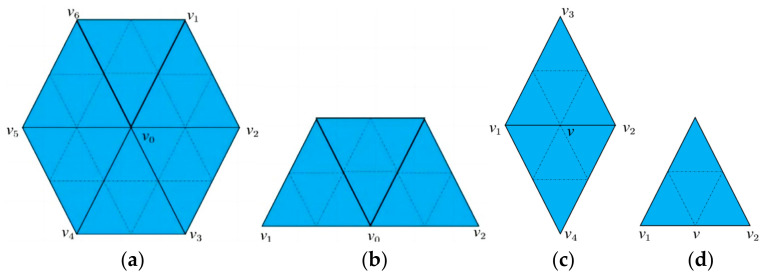
Loop Subdivision Notation Illustration: (**a**) Diagram of an original interior vertex in the mesh; (**b**) Diagram of an original boundary vertex in the mesh; (**c**) Diagram of a newly introduced interior vertex in the mesh; (**d**) Diagram of a newly introduced boundary vertex in the mesh.

The Loop subdivision algorithm is specifically designed for refining triangular meshes. The fundamental rule of this algorithm is to introduce a new vertex at the midpoint of each edge, thereby subdividing an original triangle into four smaller triangles, as illustrated in Figure 3. After the subdivision, the vertex positions are updated according to the following rules:

1. Update of Original Interior Vertices: As shown in Figure 3a, the new position of an original interior vertex is a weighted average of its pre-update position and the positions of its neighboring vertices. This can be expressed as:

(1) $$\nu =(1-n\beta )\nu_{0}+\beta \sum_{i=1}^{n}\nu_{i}\;\beta =\frac{1}{n}\left[\frac{5}{8}-{\left(\frac{3}{8}+\frac{1}{4}\cos\frac{2\pi }{n}\right)}^{2}\right]$$

Here, *v*_0_ represents the vertex before the update, and *v* represents the vertex after the update; *n* is the number of neighboring vertices connected to *v*_0_; *v_i_* denotes the neighboring vertices of *v*_0_, as shown in Figure 3a.

2. Update of Original Boundary Vertices: The new position of an original boundary vertex is determined by a weighted average of its pre-update position and the positions of its neighboring vertices along the boundary, given by:

(2) $$\nu =\frac{3}{4}\nu_{0}+\frac{1}{8}(\nu_{1}+\nu_{2})$$

where *v*_0_ is the original boundary vertex, *v*_1_ and *v*_2_ are its neighboring vertices along the boundary. as shown in Figure 3b.

3. Positioning of Newly Introduced Interior Vertices: The position of a newly introduced interior vertex is a weighted average of the positions of the two endpoints of the edge it lies on, as well as the two opposite vertices connected by that edge. This is formulated as:

(3) $$\nu =\frac{3}{8}(\nu_{1}+\nu_{2})+\frac{1}{8}(\nu_{3}+\nu_{4})$$

where *v*_1_ and *v*_2_ are the endpoints of the edge, *v*_3_ and *v*_4_ are the vertices opposite to this edge, as shown in Figure 3c. The position of a newly introduced boundary vertex in the mesh is determined by the average of the positions of the two endpoints of the edge it lies on, given by: $\nu =\frac{1}{2}(\nu_{1}+\nu_{2})$, where *v* is the newly introduced boundary vertex, and *v*_1_ and *v*_2_ are the two endpoints of the edge, as shown in Figure 3d.

This systematic approach ensures that the Loop subdivision algorithm effectively smooths and refines triangular meshes, making it highly suitable for applications in computer graphics and geometric modeling.

**Figure 4:**
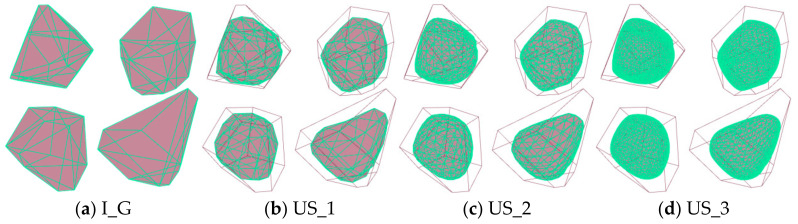
Initial Particles and Particles at Different Upsample Levels.

Figure 4 presents the morphological evolution of the four Voronoi random polyhedra introduced in Figure 2, following three successive upsampling iterations. The results clearly demonstrate that the sharp corners of the particles are subjected to the highest degree of abrasion, progressively becoming smoother with each iteration. This is followed by the edges, which also experience noticeable smoothing, while the flat surfaces of the particles exhibit the least amount of change. As the upsampling process continues, the overall shape of the particles becomes increasingly rounded, indicating a trend toward more isotropic forms. A critical observation is that, with each upsampling iteration, the number of surface meshes on the particles increases exponentially. However, this increase in mesh density is accompanied by a corresponding decrease in the extent of morphological change, suggesting that the most significant alterations occur in the initial stages of the upsampling process. This trend necessitates a balance between computational complexity and the desired level of detail in the simulation. To facilitate efficient and meaningful discrete element method (DEM) analyses, this study limits its focus to a maximum of three upsampling iterations, as further iterations yield diminishing returns in terms of shape change while significantly increasing computational costs. For clarity and consistency in the subsequent discussions, the initial particle mesh is denoted as I_G, while the meshes after the first, second, and third upsampling iterations are referred to as US_1, US_2, and US_3, respectively.

## 3. Particle Morphology Analysis

To validate the applicability of the subdivision method proposed in this study for simulating the abrasion process of river gravel, we extracted the morphological indices of coarse particles from the river source to 200 km downstream and conducted a comparative analysis with the simulated particles.

### 3.1. Particle Morphology Index System

**Figure 5:**
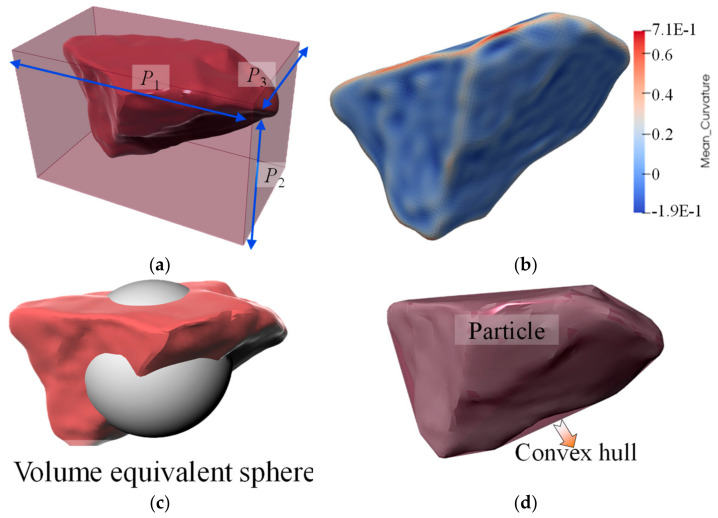
Description of Shape Index. (**a**) aspect ratio (*AR*); (**b**) mean curvature roundness index (*R*_M_); (**c**) sphericity (*S*); (**d**) convexity (*C*_X_).

Figure 5 illustrates a morphological representation method for real river particles. To measure particle morphology more scientifically, certain shape descriptors must be introduced. Due to its multi-scale characteristics, particle shape is complex to quantify. This research utilizes the particle shape quantification method proposed by Zhao and Wang [24], which is suitable for analyzing particles composed of triangular surface meshes using six shape parameters: *FI*, *EI*, *AR*, *R*_M_, *S*, and *C*_X_. The three principal directions of particles (*P*_1_ > *P*_2_ > *P*_3_), shown in Figure 5a, are determined through principal component analysis (PCA) [25,26]. The aspect ratio of a 3D particle includes the elongation index (*EI* = *P*_2_/*P*_1_) and flatness index (*FI* = *P*_3_/*P*_2_). The characteristic aspect ratio is their mean value:

(4) $$AR=\frac{EI+FI}{2}$$

Quantifying roundness requires determining the number of triangular meshes to distinguish roundness from roughness. A particle corner is defined as a part where the local mean curvature exceeds that of the particle’s maximum inscribed sphere. The mean-curvature roundness index:

(5) $$R_{M}=\sum (A_{n}\frac{k_{c}}{k_{M}})/\sum \left(A_{n}\right)$$

where *A*_n_ and *k*_M_ are the area and mean curvature of the nth triangular element, respectively, which form part of the particle’s corners, and *k_c_* is the corresponding curvature of the maximum inscribed sphere, then evaluated at these corners by the area-weighted average local mean curvature (Figure 5b).

Sphericity (*S*) and convexity (*C*_X_) are two overall shape parameters. Sphericity (*S*) indicates how close a particle is to a sphere, calculated as follows:

(6) $$S=\frac{\sqrt[{3}]{36\pi V^{2}}}{S_{A}}$$

as shown in Figure 5c, where *V* is the particle volume and *S*_A_ is the particle surface area.

Convexity (*C*_X_) quantifies the extent to which a particle approximates its convex hull, serving as an indicator of the particle’s concavity. The *C*_X_ value ranges from 0 to 1 and is calculated as follows:

(7) $$C_{X}=\frac{V}{V_{\mathrm{CH}}}$$

as shown in Figure 5d, where *V* is the particle volume and *V*_CH_ is the volume of the convex hull.

### 3.2. Particle Morphology Analysis Process

**Figure 6:**
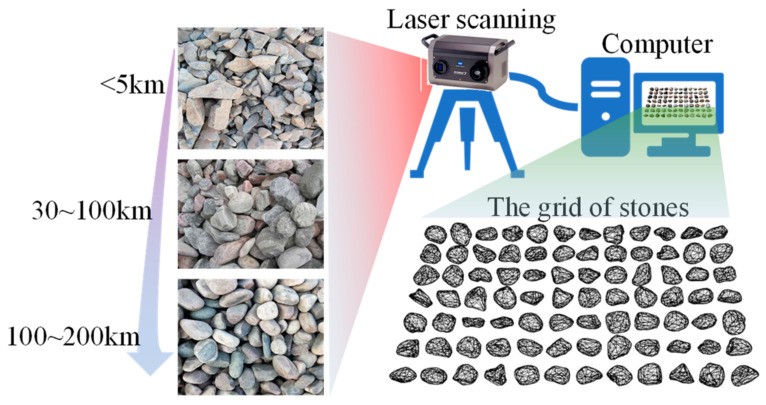
The Extraction Process of Real Particle Morphology.

The river gravel particles used in this study were sourced from northeastern Hunan Province, China, specifically from quarries located along the riverbanks between 5 km and 200 km downstream from the river’s origin. These stones were obtained using a clamshell dredging method, which maximizes the preservation of the natural integrity of riverbed stones. An average of 100 to 200 particles were selected at each sampling point. The particle samples, typically ranging in size from 10 to 60 mm, were derived from crushed and screened rock fragments, known for their irregular shapes, sharp edges, and rough surface textures. To accurately capture the true three-dimensional geometry of these particles, a non-contact 3D laser scanner, as shown in Figure 6, was employed to extract high-precision surface point cloud data of the railway ballast particles, with a scanning accuracy of up to ±10 μm. During the scanning process, the particle samples were securely placed on an automatic rotating stage. The scanner emits up to 1000 sets of laser lines, each containing over 1000 point cloud data points. As the rotating stage continuously rotates, these laser lines illuminate the entire surface of the particles and are reflected back, allowing the surface point cloud data to be transmitted and recorded on a computer. The 3D laser-scanned point cloud images of the particle samples accurately capture the geometric shape, faithfully reproducing the irregular geometry, sharp edges, and rough surface textures of the particles.

**Figure 7:**
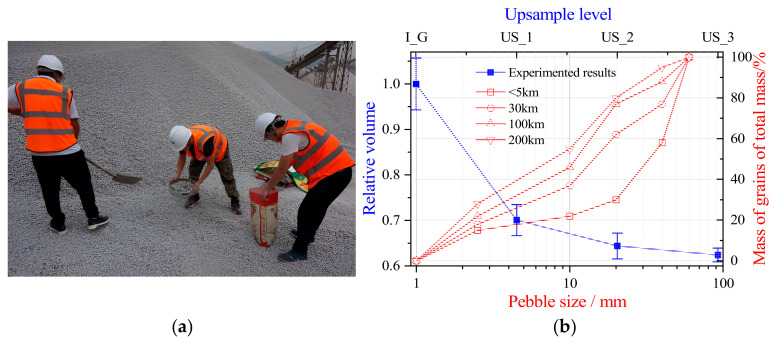
Particle Size Distribution Test. (**a**) On-site Screening and Weighing; (**b**) Particle Size Distribution at Different Upsample Levels and the Grading Distribution of Upstream and Downstream Particles.

Figure 7 illustrates the grain size distribution of particles ranging from 1 mm to 60 mm at different sampling points along the upstream and downstream riverbanks, as well as the changes in model particle volume with varying upsample levels. The upsample method proposed in this study effectively simulates the progressive refinement of particles during the abrasion process. Field observations indicate significant differences in grain size distribution between upstream and downstream regions. The downstream region exhibits a more uniform grain size distribution, primarily due to variations in water flow velocity and energy. In the upstream region, the steeper slope and faster water flow with higher energy enable the river to transport larger, heavier particles, such as gravel and pebbles. In this high-energy environment, larger particles only deposit when the flow energy decreases or encounters obstacles, leading to the formation of coarse-grained sediment. As a result, upstream sediments are typically composed of larger, rougher particles with poor sorting. As the river enters the downstream region, the slope decreases, water flow slows, and the river’s ability to transport larger particles diminishes. Consequently, smaller and lighter particles, such as fine sand, silt, and even clay, begin to deposit, typically forming thinner, more uniformly sorted sediment layers.

**Figure 8:**
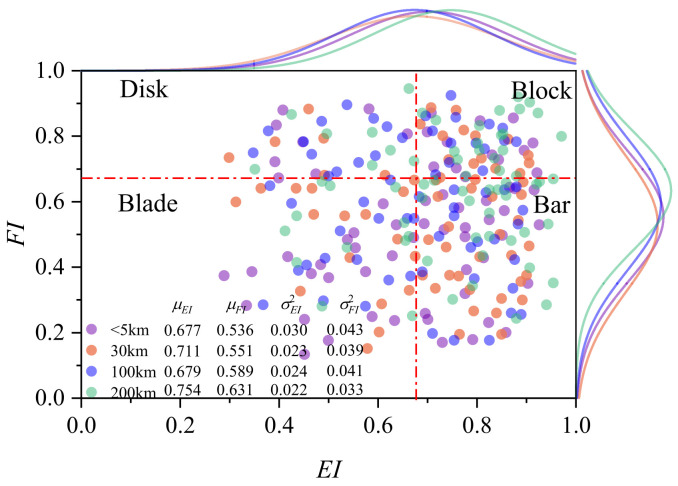
Distribution of Particle *EI* and *FI* in Different Watersheds.

Subsequently, we selected approximately 100 particles from each region within a 5 km to 200 km range from the river source and measured their *EI* and *FI*, with the results shown in Figure 8. *EI* and *FI* were used to categorize particles into four typical shapes: Disk, Block, Blade, and Bar, using a threshold of 0.67. The scatter plot results indicate that almost all particles’ *EI* and *FI* values are concentrated between 0.3 and 0.9, following a normal distribution. Here, $\mu_{EI}$ and $\mu_{FI}$ represent the mean *EI* and *FI* values of the particles, while $\sigma_{EI}^{2}$ and $\sigma_{FI}^{2}$ denote their respective variances. Overall, as the transport distance increases, the average *EI* and *FI* values also gradually increase, but no significant changes were observed in the variability of the distribution. The majority of particles fall into the Block and Bar categories, with *EI* and *FI* slightly increasing along the river course. This increase can be attributed to the high energy and turbulent flow in the upstream region, where thicker particles tend to deposit, while thinner or longer particles are more likely to be transported downstream. In the downstream region, the reduced water flow velocity and energy result in the deposition of smaller, flatter, or elongated particles. This explains why *EI* and *FI* values are higher for downstream particles, as these shapes exhibit greater stability in the water flow, making it easier for them to retain their flat or elongated forms.

**Figure 9:**
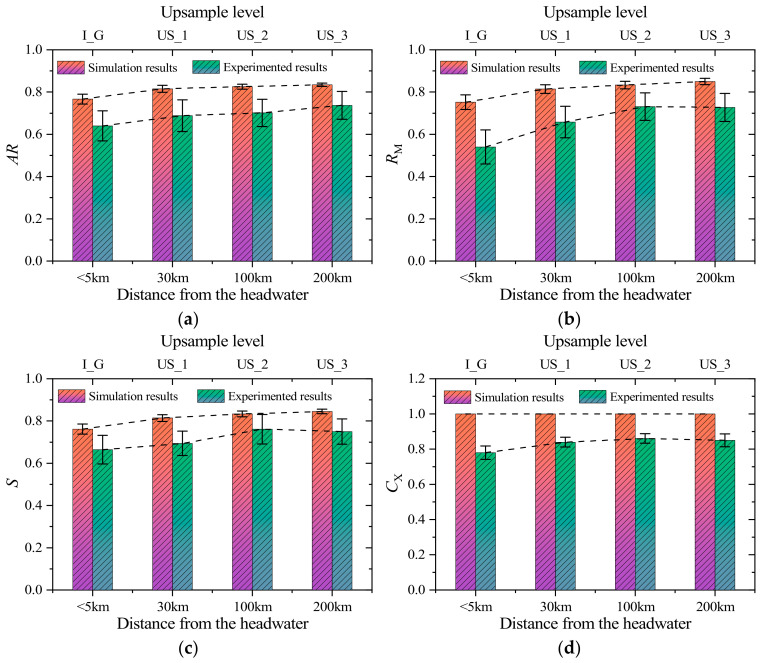
Comparison of the morphology between real and simulated particles in the upstream and downstream of the river: (**a**) aspect ratio (*AR*); (**b**) mean curvature roundness index (*R*_M_); (**c**) sphericity (*S*); (**d**) convexity (*C*_X_).

Figure 9 presents a comparative analysis of morphological indices, focusing solely on the shape change trends between simulated particles and real particles. Both simulated and observed particles display significant trends in shape evolution with increasing transport distance. Figure 9a shows that the aspect ratio of both simulated and real particles increases with transport distance, indicating a transition toward a more “block-like” shape. This phenomenon primarily occurs because, as particles are transported downstream, collisions and friction between particles gradually smooth out prominent edges, leading to a more symmetric and regular shape. Combined with the results in Figure 9c, this also suggests that particles tend to become more spherical during transport, particularly in the downstream regions where the river is flatter, and the flow velocity is lower, making this shape change more pronounced. However, this trend is not infinite, as the shapes of particles in the downstream region seem to stabilize. This is due to the more stable hydrodynamic conditions in these areas, where the interactions between particles weaken, leading to less variation in particle shape. Figure 9b,d describes the shape change patterns in terms of local morphological features, such as surface roughness. The mean-curvature roundness index of the particles shows a significant increase, indicating that sharp protrusions and surface irregularities on the particles gradually smooth out. This smoothing process is primarily due to the gradual wear of sharp edges on the particle surface caused by prolonged physical weathering and chemical erosion. Additionally, experimental results show a slight increase in particle convexity within the basin, suggesting that while the overall shape of the particles becomes more spherical, the surface roughness remains relatively unchanged. This can be attributed to the complex morphological characteristics of river particles, where some protruding parts experience only minor wear during transport. In contrast, the convexity of the simulated particles does not change with increased upsampling levels because particles generated based on the Voronoi method are inherently convex polyhedra, and the upsampling method based on Loop subdivision used in this study does not alter particle roughness.

## 4. Influence of Particle Morphology on the Mechanical Behavior of Granular Materials

### 4.1. Experimental Method

**Figure 10:**
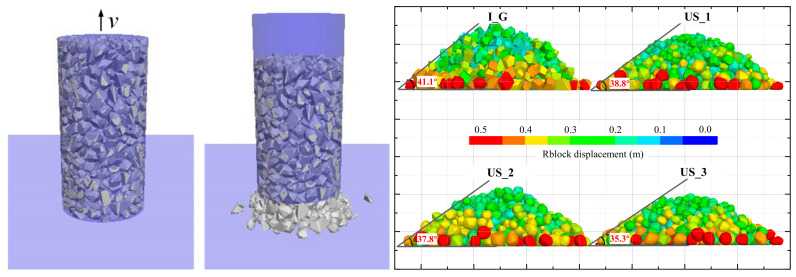
Angle of repose in discrete element method (DEM) simulations.

The angle of repose is the maximum angle of a slope at which loose particles remain stationary. For materials such as loose sand and gravel, which are composed of non-cohesive, discrete particles, the angle of repose is generally similar to the peak friction angle of the soil [27]. In this study, the angle formed by the sand heap under the belt pulley meets the conditions for free deposition and a stable state, thus it can be considered as the natural angle of repose for the sand. Based on this physical phenomenon, this study uses discrete element simulations to investigate the relationship between particle shape parameters and the internal friction angle (angle of repose). As shown in Figure 10, the numerical model randomly generates a section of gravel particles within a cylinder with a diameter of 100 mm and a height of 200 mm. It is assumed that no particle breakage occurs, and the particle contact behavior follows the linear contact model. In this model, the normal contact force is assumed to be proportional to the overlap depth between particles, while the tangential force is proportional to the shear displacement that occurs during contact. The tangential contact force adheres to Coulomb’s friction law, meaning that when the tangential contact force reaches the limit friction force, sliding occurs at the contact point. The linear contact model effectively simulates the behavior of non-cohesive particles during contact. To eliminate the influence of boundary friction on the motion of the sample, the friction coefficient between the particles and the sidewalls is set to 0, while the friction coefficient between the bottom contact surface and the particles is set to be the same as the inter-particle friction coefficient. Based on previous calibration work [19,28], the contact parameters of the numerical model are listed in Table 1. 

At the beginning of the experiment, the particles, under their self-weight, form a uniform and stable loose aggregate within the cylinder. To prevent excessive particle overlap, which could lead to boundary penetration, the linear and angular velocities of the particles are reset to zero every 100 time steps to eliminate internal unbalanced forces. Once the sample reaches a stable state, a vertical upward velocity of 1 × 10^−7^ m/step is gradually applied to simulate the slow lifting of the cylinder. Eventually, under the combined effects of gravity, particle interlocking, and friction, the upper particles slide toward the base of the pile, forming a conical shape. The stable angle formed between the conical surface and the horizontal plane can be considered the internal friction angle of the gravel material. Once the sidewalls are completely lifted and the system stabilizes over time, all 4 groups of particle materials form stable stone piles. However, with increasing upsample levels of the particles, the angle of repose of the aggregate shows a declining trend.

### 4.2. Analysis of Angle of Repose Results

**Figure 11:**
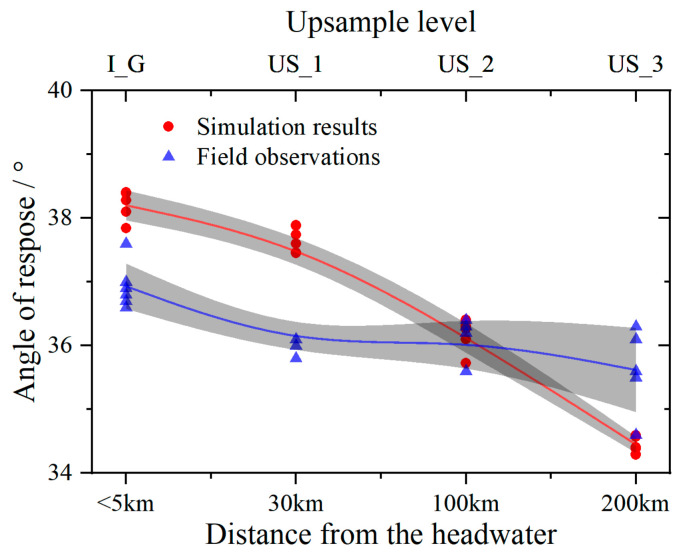
The repose angles of different watersheds and varying upsample levels.

Figure 11 compares the natural angles of repose of particle deposits from the upper and lower reaches of the river, as well as the angles obtained from the numerical method used in this study. Both sets of experiments exhibit a decreasing trend in the angle of repose with increasing distance from the headwater, although the exact slopes and curvatures of the simulation and experimental curves differ. The angle of repose obtained from the numerical simulation is slightly larger than the field observations. The results of both experiments are relatively close, with the internal friction angles of particles with different degrees of abrasion ranging from 34.4° to 38.2°. The natural angle of repose of the upstream particles is slightly larger than that of the downstream particles, while the field observation results are more scattered. The simulation method in this study effectively reproduces the interlocking effect of irregular particles, particularly angular particles, which tend to interlock during deposition, thereby increasing the stability of the deposit and making the particles less likely to slide, resulting in a larger angle of repose. In contrast, round particles lack this interlocking capability, making them more prone to rolling, which leads to a rearrangement of the particle system and ultimately results in a smaller angle of repose.

## 5. Conclusions

This study simulates the rock fracture process using the Voronoi tessellation method, followed by modeling the particle abrasion at different levels using the Loop subdivision method. The morphological distribution of real particles from upstream and downstream river sections was analyzed, and the results were compared with the morphological distribution of simulated particles at different subdivision levels. Finally, the natural angles of repose for particles with various subdivision levels were simulated using the discrete element method (DEM). The proposed simulation method was validated from both morphological and mechanical perspectives. The specific conclusion is as follows:

1. Morphological Characteristics of Upstream and Downstream Particles: This study systematically analyzed the morphological differences in river particles from upstream to downstream sections. Upstream particles exhibited more angular and irregular shapes, while downstream particles showed a gradual rounding due to continuous erosion and transport. These morphological changes highlight the varying impact of river dynamics on particle shape evolution along the river’s course.
2. Simulation of Particle Rounding with Loop Subdivision: Additionally, using the Loop subdivision method, the study effectively simulated the trend of particle rounding during transport and abrasion. The method accurately represented the smoothing of sharp edges, replicating the natural process of rounding observed in river particles over time. This modeling approach successfully captured the transition toward more spherical and isotropic particle shapes.
3. Angle of Repose Captured by Discrete Element Method (DEM): By employing the Discrete Element Method (DEM), the study accurately captured the natural angle of repose of particles with different degrees of abrasion. The results aligned closely with field observations, validating the DEM’s ability to simulate the mechanical behavior of granular materials. The findings confirmed that angular particles exhibit larger angles of repose due to interlocking, whereas rounder particles tend to slide, resulting in smaller angles.

This method can be integrated into infrastructure design near rivers to optimize materials and reduce erosion-related risks, as well as applied in floodplain management to minimize the socio-economic impacts of flooding. It also holds potential for improving efficiency and sustainability in mining by optimizing material processing, reducing energy consumption, and operational costs.

## Figures and Tables

**Table 1:** Parameters for particles in the proposed model.

Parameter	Value
Particle density, *ρ* (kg/m^3^)	2600
Particle coefficient of friction, *μ_c_*	0.5
Sidewall–particle friction *μ_s_*	0.0
Bottomplate–particle friction *μ_b_*	0.5
Particle normal stiffness, *k*_n_ (N/m)	1 × 10^8^
Particle tangential stiffness, *k*_s_ (N/m)	5 × 10^7^
Damping coefficient, *d_p_*	0.3

## Data Availability

The original contributions presented in the study are included in the article, further inquiries can be directed to the corresponding author.
